# Ellagic Acid Recovery by Solid State Fermentation of Pomegranate Wastes by *Aspergillus niger* and *Saccharomyces cerevisiae*: A Comparison

**DOI:** 10.3390/molecules24203689

**Published:** 2019-10-14

**Authors:** Federica Moccia, Adriana C. Flores-Gallegos, Mónica L. Chávez-González, Leonardo Sepúlveda, Stefania Marzorati, Luisella Verotta, Lucia Panzella, Juan A. Ascacio-Valdes, Cristobal N. Aguilar, Alessandra Napolitano

**Affiliations:** 1 Department of Chemical Sciences, University of Naples Federico II, via Cintia 4, I-80126 Naples, Italy; federica.moccia@unina.it (F.M.); panzella@unina.it (L.P.); 2 Bioprocesses and Bioproducts Research Group, Food Research Department, School of Chemistry, Universidad Autónoma de Coahuila, 25280 Saltillo, Coahuila, Mexico; carolinaflores@uadec.edu.mx (A.C.F.-G.); monicachavez@uadec.edu.mx (M.L.C.-G.); leonardo_sepulveda@uadec.edu.mx (L.S.); alberto_ascaciovaldes@uadec.edu.mx (J.A.A.-V.); 3 Department of Environmental Science and Policy, University of Milan, Via Celoria 2- 20133, Milan, Italy; stefania.marzorati@unimi.it (S.M.); luisella.verotta@unimi.it (L.V.)

**Keywords:** solid state fermentation, *Aspergillus niger*, *Saccharomyces cerevisiae*, ellagic acid, ellagitannins, ultrasound microwave-assisted extraction, acid hydrolysis

## Abstract

Fermentation in solid state culture (SSC) has been the focus of increasing interest because of its potential for industrial applications. In previous studies SSC of pomegranate wastes by *Aspergillus niger* has been extensively developed and optimized for the recovery of ellagic acid (EA), a high value bioactive. In this study we comparatively investigated the SSC of powdered pomegranate husks by *A. niger* and *Saccharomyces cerevisiae* and evaluated the recovery yields of EA by an ultrasound and microwave-assisted 7:3 water/ethanol extraction. Surprisingly enough, the yields obtained by *S. cerevisiae* fermentation (4% *w*/*w*) were found 5-fold higher than those of the *A. niger* fermented material, with a 10-fold increase with respect to the unfermented material. The EA origin was traced by HPLC analysis that showed a significant decrease in the levels of punicalagin isomers and granatin B and formation of punicalin following fermentation. Other extraction conditions that could warrant a complete solubilization of EA were evaluated. Using a 1:100 solid to solvent ratio and DMSO as the solvent, EA was obtained in 4% yields from *S. cerevisiae* fermented husks at a high purity degree. Hydrolytic treatment of *S. cerevisiae* fermented pomegranate husks afforded a material freed of the polysaccharides components that gave recovery yields of EA up to 12% *w*/*w*.

## 1. Introduction

The production of bioactives from agro-industrial by-products is nowadays a cutting-edge topic within circular economy systems and the use of biotechnological methods is one of the most actively pursued approaches. In this connection, fermentation represents an environmentally clean technology for production and extraction of such bioactives, providing high quality and high activity extracts for application as food additives or cosmetic ingredients [1,2,3,4,5].

Among fermentation techniques, solid state culture (SSC) in which the microorganisms develop on solid substrates in the lack of open liquid is one of the most widely used methods since it is of high industrial interest. The advantage of this technique is the elevated volumetric productivity with high concentrations of products reached and much reduced effluent production.

This technique has been applied to a variety of wastes produced in large amounts like rice bran, sugarcane bagasse, fruit pomaces, and peanut press cake to get mostly phenolic compounds by use of a variety of microorganisms [6,7,8,9,10].

Pomegranate is currently one of the most extensively investigated sources of health beneficial phenolics, including ellagitannins, flavonoids, anthocyanins, and other polyphenols [11,12,13,14,15]. These components are present in all parts of the fruit including peels, arils, and juice, and are responsible for a variety of biological activities that have been extensively documented [16,17,18].

Ellagic acid (EA) is the highest added value bioactive of pomegranate based on the many reports in the literature documenting anti-mutagenic and anti-carcinogenic activity [19], prevention of the onset of cardiovascular diseases, atherosclerosis, and dyslipidemic disorders [20], and stimulation of wound healing and skin elasticity [21]. By the action of gut microbiota, EA is transformed into urolithins that are effective chemopreventive and anti-inflammatory agents [22,23].

Yet, EA is present at very low levels in pomegranate, occurring mostly in the complex form of ellagitannins, including punicalagin isomers, and granatin [24,25,26] from which it can be released only following extensive hydrolysis or by biodegradation [12,27,28].

However, recovery of EA from ellagitannins presents many drawbacks including the high-production cost and the large amount of by-products generated as a result of ellagitannins degradation, making its recovery and purification difficult [15].

A promising strategy for converting ellagitannins into EA is fungal fermentation, that has been implemented in solid state systems by various strains [29,30] and particularly by *Aspergillus niger* [24,28,31] using ellagitannin rich plant materials thanks to the tannin acyl hydrolase activity of these fungi [28]. Examples include valonia tannins in axenic culture of *A. niger* SHL [32] or in co-culture of *Candida utilis* and *A. niger* [33], as well as creosote bush ellagitannins [34].

In the frame of a project aimed at exploitation of large amounts of wastes left after *Saccharomyces cerevisiae* fermentation of pomegranate fruits aided by addition of sucrose for production of wines, we found that extraction of these materials could afford EA in very good yields up to 4% (*w*/*w* with respect to the starting material) in an almost pure form [35].

On this basis in the present work we applied the solid state fermentation techniques to investigate the effects *S. cerevisiae* on pomegranate wastes as those derived from juice production in comparison with those obtained with *A. niger* whose tannase activity has already been described [28], by HPLC monitoring the formation of EA and the changes of levels of low molecular weight ellagitannins. Surprisingly enough, the yields of EA obtained by *S. cerevisiae* fermentation were found 5-fold higher than those of the *A. niger* fermented material, with a 10-fold increase with respect to the unfermented materials. Running the extraction of the fermented material with DMSO, a solvent in which EA shows a relatively high solubility, we were able to show that the yields obtained with food compatible solvents like water/ethanol mixtures do not actually reflect EA released by the action of the hydrolytic enzymes, but rather the solubility of EA in those solvents. Moreover, the effects on the recovery yields of EA of an acid hydrolytic treatment on the fermented/unfermented materials were evaluated.

## 2. Results and Discussion

### 2.1. Solid State Fermentation

Solid state fermentation of pomegranate husks was run on finely pulverized materials. For fermentation with *A. niger* the conditions that have been optimized in previous investigations were adopted [36]. A tray reactor was used, and fermentation was run at a temperature of 30 °C at 80% humidity with an inoculum level of 1 × 10^6^ spores/g.

The conditions for solid state fermentation with *S. cerevisiae* yeast were also optimized using the two-level Plackett–Burman experimental design. Fermentation was run putting the powdered material on Petri dishes taken at 25 °C and 70% humidity. The material was treated with 1 × 10^6^ spores/g inoculum, peptone 20 g/L, yeast extract 5 g/L and NaCl 460 g/L (see also Materials and Methods Section).

Following fermentation that in either case was run over 48 h, the pomegranate husk powder was oven dried and then extracted with 7:3 *v/v* ethanol/water mixture at a 1:16 solid to solvent ratio employing an ultrasound microwave system. For comparative purposes the unfermented material was subjected to same drying and extraction procedure.

### 2.2. EA Recovery

HPLC analysis was aimed at determining the levels of EA in the extracts from the fermented and unfermented pomegranate powder. An EA content of 4.8 mg/g (±0.6) was obtained for the unfermented material. This value was doubled and equal to 9.0 mg/g (±0.3) for the *A. niger* fermented material and increased 5-fold (46 mg/g (±3), that is 4.5% *w*/*w*, for the *S. cerevisiae* fermented pomegranate. The differences between the two fermentation conditions were statistically significant (*p* values < 0.05). These results are in line with those reported in our previous studies [37], confirming the rise of extractable EA following the action of *A. niger*. On the other hand, the significant increase of available EA following *S. cerevisiae* fermentation is of great interest and could have been anticipated only in part based on the high yields of recovery of EA from extraction of wastes of pomegranate wine production, though in that case sucrose was added to favor the action of the yeast [35]. These values are very interesting and well compare or are even superior to those obtained with submerged fermentation methodologies [38].

### 2.3. Identification and Quantitative Analysis of Ellagitannins

To get an insight into the effects of fermentation of the ellagitannins present in the pomegranate husks under the two conditions investigated, and to evaluate the mode of generation of EA, the extracts were subjected to qualitative analyses by LC/MS. Table 1 reports the ellagitannin components of the extracts of pomegranate husks before and after fermentation.

As expected, punicalagin α and β are present in the fresh pomegranate wastes as well as in the material derived from fermentation of *A. niger* though at different levels. For *S. cerevisiae* fermented material only punicalagin α could be detected. EA was found in all the extracts. A number of other ellagitannins were also detected that have been previously described as components of pomegranate ellagitannins or generated by acid hydrolysis of high molecular weight species [12,25,39].

**Figure 1:**
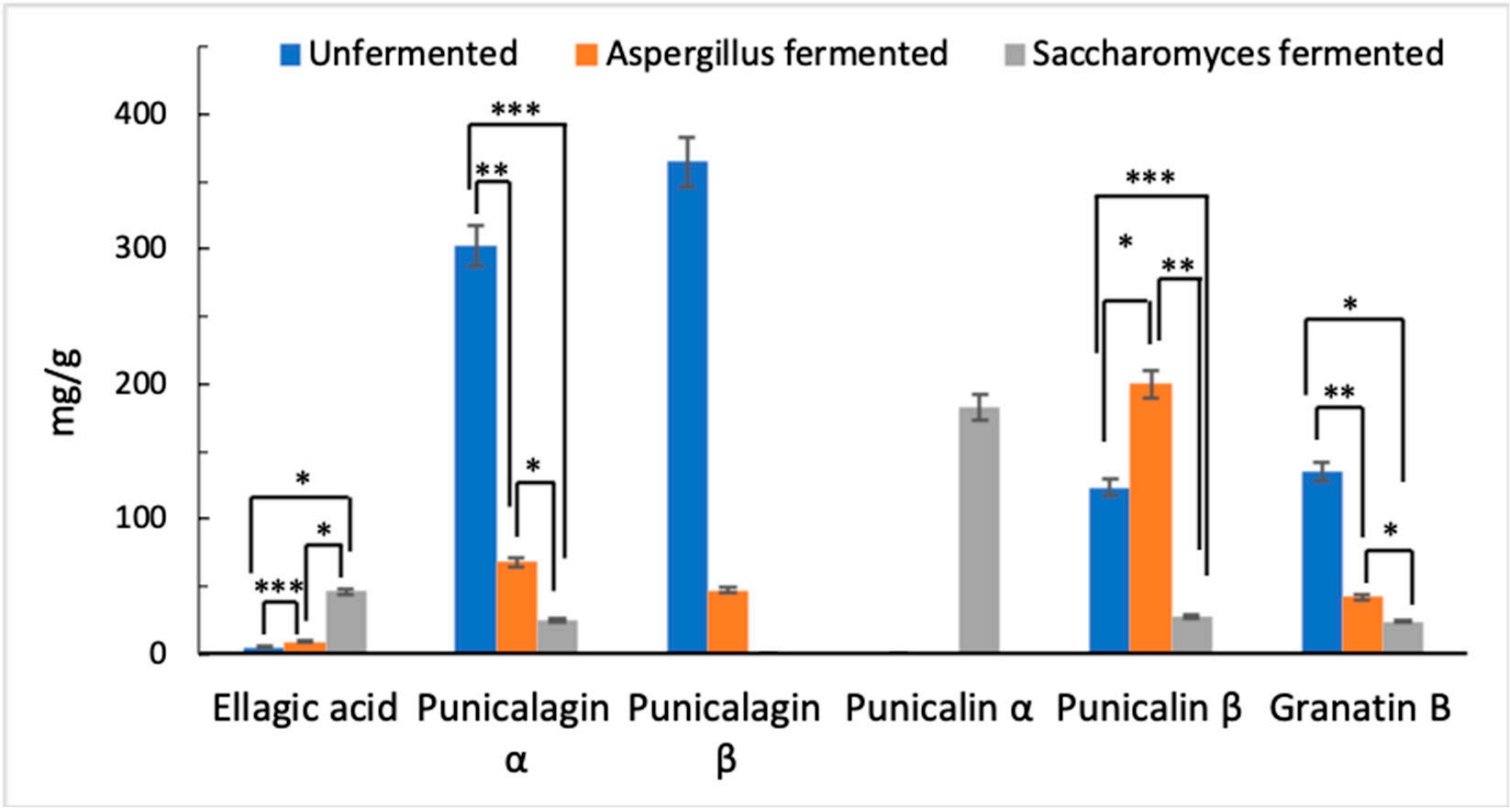
Quantitative analysis of the main ellagitannins identified in the fermented/unfermented pomegranate husks extracts. Statistical analysis is shown on the bars: * *p* < 0.05; ** *p* < 0.01; *** *p* < 0.005.

Quantitative analysis of the main components was then run on the unfermented and fermented materials. Data in Figure 1 show high levels of punicalagin α and β for the unfermented material, relatively high levels of punicalin β and granatin B. Interestingly, statistically significant differences are apparent for the fermented material vs. the unfermented material and for the fermented materials under the two conditions investigated.

**Scheme 1:**
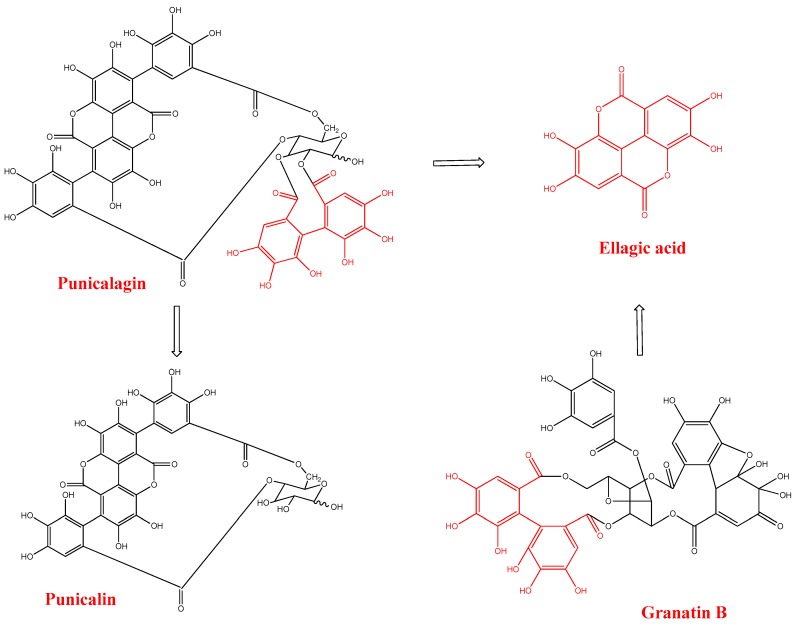
Structures of the main ellagitannins identified and likely formation pathways of ellagic acid (EA).

It is known that hydrolytic cleavage of punicalagin α and β at the sugar moiety leads to punicalin α/β and hexahydroxydiphenic acid (HHDP), this latter then converted to EA by spontaneous lactonization (Scheme 1).

Indeed, for the *Aspergillus* fermented pomegranate, the punicalagin levels drop down, though the levels of punicalin β do not rise accordingly. The levels of granatin B that may give rise to ellagic acid by a different hydrolytic pathway (Scheme 1) are lowered with statistical significance after *A. niger* fermentation to around one fourth of the value obtained for the unfermented material.

On the other hand, for the *S. cerevisiae* fermented material, punicalagin α is drastically reduced while β isomer is below detection limits. Here again the punicalin α and β levels vary with respect to the unfermented material, with punicalin α well detectable differently from what observed in *A. niger* fermentation. The decrease of granatin B levels is more pronounced for the *S. cerevisiae* fermentation with respect to *A. niger* fermentation.

On this basis some conclusions can be drawn:

- Punicalagins α/β represent the main target of the hydrolase activity of the microorganisms suggesting a likely pathway of formation of EA.
- The occurrence of punicalin further confirms the hydrolytic cleavage of punicalagin during fermentation as a reaction pathway responsible at least in part for the generation of EA.
- Hydrolysis of granatin B may have a central role in the generation of EA during fermentation.
- Besides *A. niger* fungus, *S. cerevisiae* yeast is also capable of affecting the hydrolysis of ellagitannins to EA leading to even higher yields of the compound.

This latter issue is of particular interest as it confirms the presence of tannase activity of *S. cerevisiae* that has only recently been identified and characterized [40].

In addition, these data would suggest that fermented pomegranate wastes containing high levels of EA as a result of the fermentation/hydrolytic process can exert health beneficial effects.

### 2.4. Evaluation of Different Extraction Conditions

In further experiments we explored different conditions for extraction of EA from the fermented materials against the starting pomegranate wastes. Consideration of the poor solubility of EA in alcohols and water led us to use DMSO in which the compound exhibits the highest solubility to evaluate the extraction recovery. The investigation was restricted to the Saccharomyces fermented materials given the higher recovery yields of EA obtained.

A yield of 5.9 mg/g (±0.4) was obtained for the unfermented material using a solid to solvent ratio of 1:10 g/mL under stirring for 1 h, whereas the fermented material gave a 21 mg/g (±1.2) yield. To assess whether the EA yields obtained by DMSO extraction could be still limited by the low solubility, the fermented material was repeatedly extracted, and the amount of EA recovered after each run determined. It was found that each extraction afforded some additional 5 mg/g yield up to an overall yield of 30 mg/g (±1) (from three extraction steps). Use of a much lower solid to solvent ratio (1:100 g/mL) resulted in an increase of the recovery yield to 40 mg/g (±0.2), that is a 4% *w*/*w* yield comparable to that obtained with the ultrasound microwave assisted extraction used in the comparative analysis described above. On the other hand, using the 7:3 ethanol:water mixture but without the ultrasound and microwave aid the yield of EA was only 2 mg/g. This result indicates that the solubility of EA into the extraction solvent may represent a severe limitation for the scale up of this procedure in the perspective of an extensive exploitation of pomegranate wastes.

**Figure 2:**
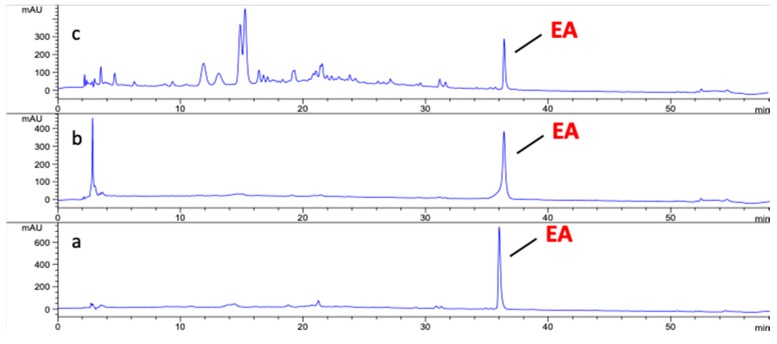
HPLC elution profile of the extracts of *Saccharomyces cerevisiae* fermented pomegranate wastes obtained using DMSO (**a**) (diluted 1:20), ethanol (**b**) (diluted 1:5), and water (**c**) (diluted 1:5) at a solid to solvent ratio of 1:10 g/mL.

HPLC analysis of the DMSO extracts with detection wavelength set at 254 nm showed a very clean chromatographic profile with EA as the main if the not the sole component (Figure 2a). This could be explained considering that differently from water (Figure 2c) or alcohol/water mixtures that extract also more polar ellagitannin containing glucose moieties, this solvent is rather selective for EA. Using pure ethanol (profile b) the extraction of EA is selective to an extent comparable to that observed for DMSO, but the recovery yields are much lower (4 mg/g ± 0.1) because of the poorer solubility of EA in this solvent.

### 2.5. Effects of Acid Hydrolysis on EA Recovery

Based on our previous studies on plant waste materials including spent coffee grounds and residues of pomegranate wine production showing that an acid hydrolytic treatment induced efficient removal of the polysaccharides components and resulted in an enhancement of the activities of the polyphenolic components [35,41], we applied such protocol to the *S. cerevisiae* fermented pomegranate wastes and the unfermented material for comparison. The resulting materials were then extracted with DMSO using a 1:100 g/mL solid to solvent ratio. The recovery yields of EA from unfermented and fermented pomegranate were 91 mg/g (±3) and 120 mg/g (±7), respectively. When corrected for the loss of weight associated to the acid treatment, values of 150 and 123 mg/g could be estimated. This would indicate that the fermentation treatment had already produced an efficient hydrolysis of the ellagitannin components leading to EA and hence the recovery yields were not changed significantly, whereas clearly this was not the case for the rough unfermented material. Additionally, this result indicates a straightforward way to get a material enriched in phenolic components from which EA recovery could be obtained in significantly high yields.

## 3. Materials and Methods

### 3.1. Microorganisms

The *S. cerevisiae* strain and *A. niger* GH1 strain from the Food Research Department collection of Autonomous University of Coahuila were used. Spores of *A. niger* were preserved in cryoprotective solution (glycerol and skimmed milk).

### 3.2. Raw Material

Pomegranate fruits were obtained from Cuatrociénegas, Coahuila, México. The husk was separated from the fruit and dehydrated at 60 °C for 48 h. Samples were pulverized to a 30-mesh particle size in an industrial homogenizer (5 L; model LP12 Series 600-182, JR Maquinaria para mercado S.A. de C.V., México). Pomegranate husk powder was stored in plastic black bags until use. Prior to establish the culture, pomegranate powder was dried, and taken to constant dry weight.

### 3.3. SSC with Aspergillus Niger

The SSC was performed on a tray bioreactor with pomegranate husk powder as the support. The fermentation time was 48 h optimal condition was temperature (30 °C), inoculum (1 × 10^6^ spores/g), humidity (80%), and NaNO_3_ (15.6 g/L), KH_2_PO_4_ (3.04 g/L), KCl (1.52 g/L), MgSO_4_ (1.52 g/L) of culture medium. These conditions were in accord with those previously reported [36].

### 3.4. SSC with Saccharomyces Cerevisiae

The SSC was carried out in Petri dishes where the pomegranate husk powder was inoculated with the yeast strain. The fermentation conditions were defined according to the two-level Plackett–Burman experimental design as shown in Table 2. All treatments were done in triplicate. The independent factors were defined as temperature (°C), humidity (%), inoculum (cells/g), pH, peptone (g/L), yeast extract (g/L), and NaCl (mg/L). The best solid state fermentation conditions were temperature 25 °C, humidity 70%, inoculum 1 × 10^6^, pH 5, peptone 20 g/L, extract of yeast 5 g/L, and NaCl 460 g/L.

### 3.5. Extraction Conditions

The fermented materials were oven dried at 50 °C until constant weight and extracted with ethanol/water (70/30) using a 1:16 mass/volume ratio. An ultrasound and microwave-assisted extraction was performed (hybrid technology system Ultrasound/Microwave Cooperative Workstation, Nanjing ATPIO Instruments Manufacture, Nanjing, China). Ultrasound time was taken at 20 min (25 KHz) and microwave frequency was set at 2450 MHz (70 °C). After this process the extracts were subjected to conventional filtration before HPLC analysis.

In other experiments different extraction procedures were run using ethanol, water, or DMSO as the solvent at a 1:10 g/mL or 1:100 g/mL ratio. In some cases, the powder was grounded with the solvent in a glass to glass homogenizer. When required, the extraction was repeated three times and the concentration of EA in the combined extracts was determined by HPLC analysis under the conditions previously reported [35].

### 3.6. Acid Hydrolysis

Fermented/unfermented pomegranate husk powder (0.3 g) was treated with 7 mL of 6 M HCl under stirring, at 100 °C, for 1 h. After cooling at room temperature, the mixture was centrifuged (7000 rpm, 30 min) and the precipitate washed with water till neutrality and freeze-dried to give a dark powder, in yields of 0.024 and 0.080 g for the unfermented and *S. cerevisiae* fermented material, respectively.

### 3.7. HPLC Analysis

Ellagic acid was quantified by HPLC/MS analysis using a commercial sample (Sigma-Aldrich, Darmstadt, Germany) as standard. Other components of the extracts were also identified and quantified against available standards. The analyses by Reverse Phase-High Performance Liquid Chromatography were performed on a Varian HPLC system including an autosampler (VarianProStar 410, Santa Clara, CA, USA), a ternary pump (VarianProStar 230I, Santa Clara, CA, USA), and a PDA detector (VarianProStar 330, Santa Clara, CA, USA). A liquid chromatograph ion trap mass spectrometer (Varian 500-MS IT MassSpectrometer, Santa Clara, CA, USA) equipped with an electrospray ion source also was used. Samples (5 µL) were injected onto a Denali C18 column (150 × 2.1 mm, 3 µm, Grace, MD, USA). The oven temperature was maintained at 30 °C. The eluents were formic acid (0.2%, *v*/*v*; solvent A) and acetonitrile (solvent B). The following gradient was applied: initial, 3% B; 0–5 min, 9% B linear; 5–15 min, 16% B linear; 15–45 min, 50% B linear. The column was then washed and reconditioned. The flow rate was maintained at 0.2 mL/min and elution was monitored at 245, 280, 320, and 550 nm. The whole effluent (0.2 mL/min) was injected into the source of the mass spectrometer, without splitting. All MS experiments were carried out in the negative mode [M−H]^−^. Nitrogen was used as nebulizing gas and helium as damping gas. The ion source parameters were spray voltage 5.0 kV and, capillary voltage and temperature were 90.0 V and 350 °C, respectively. Data were collected and processed using MS Workstation software (V 6.9). Samples were analyzed in full scan mode acquired in the *m*/*z* range 50–2000. In other experiments analyses of extracts from fermented/unfermented materials were run under the conditions reported previously [35].

### 3.8. Statistical Analysis

Data sets were initially assessed for normality. Means were statistically compared using independent samples two-tailed ANOVA tests (Microsoft Excel 2010, Redmond, WA, USA).

## 4. Conclusions

Solid state culture (SSC), a methodology with a great potential for industrial applications, has been widely applied to fermentation by fungi, particularly by *A. niger*, of different tannin-rich vegetable sources and agri-food wastes [34,36,37].

In the present study this methodology was applied to *S. cerevisiae* yeast using pomegranate husk powder as support. An efficient hydrolysis of EA-releasing tannins particularly punicalagin α and β as well as granatin B leading to EA was observed and the yields obtained by extraction with a food compatible ethanol/water mixture aided by combined ultrasound and microwave treatment (4% *w*/*w*) proved 5-fold higher than those obtained with *A. niger* and 10-fold higher than those of the unfermented material under the same extraction conditions. Moreover, the possibility to improve the yeast activity by addition of a saccharose source, possibly an agro-industrial by-product, represents an interesting perspective to be investigated.

The poor solubility of EA in most of the extraction conditions commonly used has a negative impact on the recovery yields. The use of DMSO, a solvent ensuring a good solubility of the acid, allowed to assess that the recovery yields of EA are severely limited by the low solubility of this compound in most of the extraction conditions. Yet, such limitation could be overcome by the use of the combined ultrasound and microwave methodology. A material with high recovery yields of EA could be obtained by a hydrolytic treatment of the fermented pomegranate husks allowing efficient removal of polysaccharides.

With their high levels of available EA, yeast fermented pomegranate wastes have a potential as food supplement and could exert health beneficial effects possibly even superior to those of fresh fruit extracts that contain mainly punicalagin and other high molecular weight tannins with low bioavailability profile.

## Figures and Tables

**Table 1:** Identification of ellagitannin-related compounds in the extracts of unfermented/fermented pomegranate husks powder by LC/MS analysis.

		Compound		
Retention Time (min)	[M−H]^−^	Unfermented Material	Fungal Fermentation	Yeast Fermentation
4.55	377.1	3,4-DHPEA-EA ^a^	3,4-DHPEA-EA ^a^	
4.75	781.1			Punicalin α
15.47	781.1	Punicalin β	Punicalin β	Punicalin β
18.07	783.1	Terflavin B	Terflavin B	
21.93	1083	Punicalagin α	Punicalagin α	Punicalagin α
22.58	1083	Punicalagin β	Punicalagin β	
27.99	633.1	Galloyl-HHDP-hexoside ^b^		
28.67	463.1	Ellagic acid-hexoside		
29.83	784.1	Pedunculagin II		Pedunculagin II
30.27	934.9			Casuarinin
31.22	951	Granatin B	Granatin B	Granatin B
32.74	433	Ellagic acid pentoside		
33.76	301	Ellagic acid	Ellagic acid	Ellagic acid

^a^ (3,4-DHPEA-EA): 3,4-dihydroxyphenylethanol-elenolic acid; ^b^ (HHDP): hexahydroxydiphenic acid.

**Table 2:** Plackett–Burman experimental design for the evaluation of solid state culture (SSC) conditions of pomegranate residues with *Saccharomyces cerevisiae*.

Treatment	T (°C)	Humidity (%)	Inoculum (cells/g)	pH	Peptone (g/L)	Extract of Yeast (g/L)	NaCl (g/L)	EA (mg/g)
1	−1	−1	−1	1	1	1	−1	0.15
2	1	−1	−1	−1	−1	1	1	0.03
3	−1	1	−1	−1	1	−1	1	0.65
4	1	1	−1	1	−1	−1	−1	0.08
5	−1	−1	1	1	−1	−1	1	0.11
6	1	−1	1	−1	1	−1	−1	0.14
7	−1	1	1	−1	−1	1	−1	0.09
8	1	1	1	1	1	1	1	0.11
9	−1	−1	−1	1	1	1	−1	0.26
10	1	−1	−1	−1	−1	1	1	0.27
11	−1	1	−1	−1	1	−1	1	0.33
12	1	1	−1	1	−1	−1	−1	0.21
13	−1	−1	1	1	−1	−1	1	0.06
14	1	−1	1	−1	1	−1	−1	0.15
15	−1	1	1	−1	−1	1	−1	0.07
16	1	1	1	1	1	1	1	0.31
17	−1	−1	−1	1	1	1	−1	0.20
18	1	−1	−1	−1	−1	1	1	0.29
19	−1	1	−1	−1	1	−1	1	0.26
20	1	1	−1	1	−1	−1	−1	0.37
21	−1	−1	1	1	−1	−1	1	0.26
22	1	−1	1	−1	1	−1	−1	0.27
23	−1	1	1	−1	−1	1	−1	0.18
24	1	1	1	1	1	1	1	0.36
